# Dysregulation of Protease and Protease Inhibitors in a Mouse Model of Human Pelvic Organ Prolapse

**DOI:** 10.1371/journal.pone.0056376

**Published:** 2013-02-20

**Authors:** Madhusudhan Budatha, Simone Silva, Teodoro Ignacio Montoya, Ayako Suzuki, Sheena Shah-Simpson, Cecilia Karin Wieslander, Masashi Yanagisawa, Ruth Ann Word, Hiromi Yanagisawa

**Affiliations:** 1 Department of Molecular Biology, University of Texas Southwestern Medical Center, Dallas, Texas, United States of America; 2 Department of Obstetrics and Gynecology, University of Texas Southwestern Medical Center, Dallas, Texas, United States of America; 3 Department of Molecular Genetics, University of Texas Southwestern Medical Center, Dallas, Texas, United States of America; 4 Howard Hughes Medical Institutes, University of Texas Southwestern Medical Center, Dallas, Texas, United States of America; Stanford University, United States of America

## Abstract

Mice deficient for the fibulin-5 gene (Fbln5^−/−^) develop pelvic organ prolapse (POP) due to compromised elastic fibers and upregulation of matrix metalloprotease (MMP)-9. Here, we used casein zymography, inhibitor profiling, affinity pull-down, and mass spectrometry to discover additional protease upregulated in the vaginal wall of *Fbln5^−/−^* mice, herein named V1 (25 kDa). V1 was a serine protease with trypsin-like activity similar to protease, serine (PRSS) 3, a major extrapancreatic trypsinogen, was optimum at pH 8.0, and predominantly detected in estrogenized vaginal epithelium of *Fbln5^−/−^* mice. PRSS3 was (a) localized in epithelial secretions, (b) detected in media of vaginal organ culture from both *Fbln5^−/−^* and wild type mice, and (c) cleaved fibulin-5 in vitro. Expression of two serine protease inhibitors [*Serpina1a* (α1-antitrypsin) and *Elafin*] was dysregulated in *Fbln5^−/−^* epithelium. Finally, we confirmed that *PRSS3* was expressed in human vaginal epithelium and that *SERPINA1* and *Elafin* were downregulated in vaginal tissues from women with POP. These data collectively suggest that the balance between proteases and their inhibitors contributes to support of the pelvic organs in humans and mice.

## Introduction

Prolapse of the pelvic organs (i.e., vagina, uterus, bladder, and rectum) represents failure of a complex dynamic system of pelvic floor support. Results obtained in our laboratories, together with the phenotype of lysyl oxidase-like 1 (Loxl1) null mice [1], [2], have led us to propose that pelvic organ prolapse (POP) is caused by altered balance between matrix synthesis, particularly elastic fibers, and protease activation. For example, mice deficient in *Fbln3* or *Loxl1* develop mild defects in elastic fibers postnatally [3], [4] and vaginal matrix metalloprotease (MMP)-9 is activated with aging or after parturition. *Fbln5* knockout mice, which fail to assemble elastic fibers, show marked upregulation of MMP-9 in the vaginal wall several weeks before the onset of prolapse [5]. In contrast, normal elastic fibers in the vaginal wall of wild type mice seem to protect these animals from proteases that are activated with ovariectomy [6], mechanical distention [7] or after parturition [8]. In the vaginal wall of *Fbln5^RGE/RGE^* knock-in mice in which the integrin binding domain of fibulin-5 is mutated, MMP-9 is also upregulated, yet these mice are protected from prolapse due to normal elastic fibers unless challenged with lysyl oxidase inhibitors to block new elastic fiber synthesis [9]. These results, together with experimental results showing protease activation in the vaginal wall of women with POP [9] suggest that protease activation is important in the pathogenesis of urogenital prolapse.

The importance of vaginal MMP-9 in the development of prolapse in *Fbln5^−/−^* mice has been confirmed. Specifically, whereas >90% of *Fbln5^−/−^* females develop prolapse by 20 weeks of age, only 40% develop prolapse if deficient in both *Fbln5* and *Mmp9* [9]. The failure of complete rescue in the double knockout mice suggests the possibility that proteases other than, or in addition to, MMP-9 may be involved. Here, we sought to identify a potential protease(s) involved in the development of POP. We discovered a serine protease of 25 kDa that was upregulated in prolapsed vaginal tissues. The balance between serine proteases and their inhibitors expressed in specific cell types of the vaginal wall was then examined in detail in both humans and mice, with or without POP. The results indicate that (i) a PRSS-3-like trypsinogen is expressed in the vaginal epithelium of *Fbln5^−/−^* mice and humans, (ii) its activity in the prolapsed vaginal wall is primarily regulated by serine protease inhibitors, and (iii) PRSS3 catalyzes degradation of fibulin-5 in vitro.

## Materials and Methods

### Mice

*Fbln5^−/−^* mice were previously described [9], [10] and kept on a 12 h/12 h light/dark cycle. All animal experimental procedures were reviewed and approved by the Institutional Animal Care and Use Committees of the University of Texas Southwestern Medical Center.

### Casein Zymography

Vaginal tissues were pulverized and homogenized in Tris buffer (10 mM Tris-HCl, pH 7.4, 150 mM NaCl, 10 mM CaCl_2_) containing 0.1% Triton-X 100. Homogenates were centrifuged at 10,000 g for 15 minutes at 4°C. Protein concentration in supernatants was determined using a Bradford protein assay kit (Bio-Rad, Hercules, CA, USA). Protein samples were prepared by mixing with nonreducing sample loading buffer without boiling.Vaginal extracts subjected to SDS-PAGE on 10% non-reducing gels containing 1 mg/ml casein hammersten (Fisher Scientific, Pittsburgh, PA, USA) and processed for zymogram analysis. After electrophoresis, the gels were washed with 2.5% (v/v) Triton X-100 followed by distilled water and incubated in zymogram developing buffer (Invitrogen, Grand Island, NY, USA) 16–18 h at 37°C. Gels were stained in 30% (v/v) methanol, 10% (v/v) acetic acid, 0.5% Coomassie Brilliant Blue R-250 and destained with 20% (v/v) methanol in 7% (v/v) acetic acid. Caseinolytic activity of 25 kDa by analysis of zymography was quantified by measuring the inverted band intensity by Image J software where appropriate.

### Effect of Inhibitors and pH on Caseinolytic Activity

Proteinase inhibitors for metalloproteinase (EDTA), serine protease (PMSF, TLCK, TPCK) were added to the samples before SDS-PAGE. For pH optimum determination, buffers were prepared ranging between pH 4 to 11 and caseinolytic activity was measured as a function of pH.

### Fluorescent Substrate Assays for Caseinolytic Activity

Caseinolytic activity was measured by EnzChek Protease assay kit (Molecular Probes, Eugene, OR, USA) according to manufacturer’s instructions. Briefly, vaginal extracts (2–4 µg) from WT or *Fbln5^−/−^* vagina were incubated with fluorogenic substrates with or without serine protease inhibitors (PMSF, TLCK, TPCK), serine/cysteine protease inhibitor (Leupeptin), aspartic protease inhibitor (Pepstatin A), or cycteine protease inhibitor (E-64) and incubated for 1 h, then fluorescence intensity was measured using microplate plate reader (Safire 2, Tanika). Experiments were performed in triplicate and repeated two times.

### Ovariectomy (OVX) and Estrogen (E2) Injections

Mice were ovariectomized at two months of age. Two weeks after ovariectomy, animals were treated with 17β-estradiol (50 µg/kg/d) for 48 h. Ovariectomized controls were injected with vehicle alone. Tissues were dissected, immediately frozen in liquid nitrogen, and stored at −80°C until casein zymography was performed.

### Tissue Acquisition and Processing

Vaginal tissues were obtained from a bank of specimens from the female reproductive tract maintained by the Department of Obstetrics and Gynecology under the approval of the Institutional Review Board at the University of Texas Southwestern Medical Center. Vaginal tissue was obtained from women undergoing hysterectomy for benign gynecologic conditions other than POP and from women having pelvic reconstructive surgery for POP (Table I). After cross-clamping of the vaginal apex and removal of the uterus, a full-thickness tissue specimen was obtained from the vaginal apex of the anterior and/or posterior vaginal wall. For patients undergoing colpocleisis procedures, the vaginal apex was identified and a tissue specimen containing both vaginal epithelium and muscularis was removed. Women with conditions known to be associated with high metalloproteinase activity (i.e., severe endometriosis/adenomyosis, rheumatoid arthritis, or cervical neoplasias) were excluded. For this study, 6 of 10 tissues specimens from the unaffected compartment (all Stage 0–1 of the posterior wall) of postmenopausal women with prolapse were considered postmenopausal controls. Women with POP were staged using the POP quantification scoring system [11]. Women in the control group underwent preoperative evaluation, including a pelvic examination to evaluate for the presence of prolapse, but formal staging was not performed. Vaginal muscularis was dissected free of vaginal epithelium (surgical mucosa) and was either snap frozen in liquid nitrogen or fixed in RNA-Later® (Ambion Technologies, Austin, TX).

**Table 1:** Demographics of tissue donors.

	Premenopausal		Postmenopausal^δ^	
Status	Control	Prolapse	Control	Prolapse
n	17	15	10	46
Age (mean ± SEM)	40.1±1.7	37.1±2.1	63.6±3.7	70.7±1.3
Vaginal parity (median,range)	2 (0,6)	3 (2,6)	3 (1,6)	3 (0,14)*
BMI	30.3±1.5	35.7±3.3	26.9±1.0	27.3±0.9**
Race				
H, n (%)	7 (41)	3 (20)	3 (30)	19 (33)
B, n (%)	7 (41)	3 (20)	0	4 (7)
W, n (%)	1 (6)	8 (53)	6 (60)	33 (57)
Other/Unknown, n (%)	2 (12)	1 (7)	1 (10)	2 (3)
Stage of sampledcompartment				
0–1	17 (100)	–	10 (100)	–
2	–	12 (80)	–	12 (26)ť
3	–	2 (13)	–	13 (28)
4	–	1 (7)	–	21 (46)

δ4 subjects with tissues from prolapsed (anterior) and supported (posterior) vaginal wall.

* P<0.05 compared with premenopausal controls.

** P<0.05 compared with premenopausal prolapse.

ť P<0.05, Chi-Square test.

### Real-Time Quantitative Polymerase Chain Reaction *(qPCR)*

qPCR was used to determine the relative levels of mRNAs in vaginal tissues of indicated genotypes or in human vaginal tissues. Mouse tissues were pulverized and homogenized in Trizol reagent (Invitrogen). RNA was isolated according to manufacturer’s protocol. In the case of human tissues, tissues were minced and homogenized in 4 M guanidinium isothiocyanate buffer and layered over 5.7 M cesium chloride and centrifuged overnight at 237,000×g to extract RNA. cDNA synthesis was carried out with 2 µg of total RNA in a reaction volume of a 20 µl. Each reaction contained 10 mM dithiothreitol (DTT), 0.5 mM deoxynucleotide triphosphates (dNTPs), 0.015 µg/µl random primers, 40 U RNase inhibitor (Invitrogen) and 200 U reverse transcriptase (Invitrogen). PCR reactions were carried out in the ABI Prism 7000 sequence-detection system (Applied Biosystems, Foster City, CA, USA). The reverse transcription product from 50 ng RNA was used as template, and reaction volumes (30 µl) contained Master Mix (Applied Biosystems). Primer concentrations were 900 nM. Cycling conditions were: 2 min at 50°C, followed by 10 min at 95°C; then 40 cycles of 15 sec at 95°C, and 1 min at 60°C. SYBR Green was used for amplicon detection. Gene expression was normalized to expression of the housekeeping gene GAPDH. A preprogrammed dissociation protocol was used after amplification to ensure that all samples exhibited a single amplicon. Levels of mRNA were determined using the ddCt method (Applied Biosystems) and expressed relative to an external calibrator present on each plate. In the case of PRSS amplification, human fetal pancreas was used as the positive control. Primers are listed in Supplemental Table I.

### Activity-based Protein Profiling of Serine Proteases

Vaginal tissue homogenates were made in tissue protein extraction reagent (T-PER) (Thermo-Scientific, Waltham, MA, USA) and centrifuged at 16,000×g at 4°C for 5 min. Protein concentration was determined by the Bradford method. Homogenate were diluted to 2 mg/ml and 50 µl (100 µg) homogenate was labeled with 1 µl of TAMRA-FP (2 µM) labeled serine hydrolase probe (Thermo-Scientific). Incubation was carried out for 60 min and then reaction stopped by adding 2× Laemmli reducing sample buffer. Labeled proteins analyzed by SDS-PAGE followed by fluorescence gel scanning. To determine the specificity of the TAMRA-FP probe, homogenates were boiled for 5 min in sample buffer before adding the probe. For activity-based pull down assays, 200 µg of homogenate was incubated with FP-biotin (2 µM (kind gift from Prof. Benjamin F Cravatt, The Scripps Research Institute, La Jolla, CA, USA) for 60 min, then streptavidin magnetic beads (50 µl, Invitrogen) were added to the reaction. After beads and supernatants were separated magnetically, beads were subjected to SDS-PAGE followed by silver staining (Invitrogen). Differential band intensity at approximately 25-kDa was quantified by Image J software. After quantification, the 25-kDa band was excised and subjected to mass spectrometric analysis by the Protein Chemistry Technology Core at the UT Southwestern Medical Center. MASCOT search program was used to identify peptide fragments.

### In vitro Cleavage of Fibulin-5

Human recombinant fibulin-5 (R&D System, Minneapolis, MN, USA) incubated with human recombinant PRSS3 at 1∶20 ratio in Invitrogen developing buffer for 2 h. Samples were boiled with reducing sample buffer and run on 4–15% gradient gel and silver stained (Invitrogen).

### Immunohistochemistry

Vaginal tissues were dissected from mice, OCT embedded and frozen in liquid N2. Frozen vaginal tissue sections (10 µm) were mounted on glass slides and brought to RT before proceeding for staining. Specimens were fixed in 4% PFA for 10 min and rinsed in PBS, blocked with 5% normal donkey serum for 1 h at RT. Specimens were incubated with PRSS3 antibody (R&D Systems) overnight at 4°C and detected with Alexa Fluor 488-donkey anti-goat secondary antibodies (1∶200).

### Vagina Organ Culture

Vagina was dissected from mice and rinsed in HBSS solution and three to five vaginas were pooled and used in culture. Free floating vaginal tissues cultured in initial HBSS solution for 72 h without antibiotic at 37 °C in the presence of CO2. Conditioned media were collected and briefly centrifuged to exclude free floating epithelial cells and protein concentration was determined.

### Immunoblot Analysis

Condition media from vaginal organ culture were subjected to 10% SDS-PAGE. Proteins were transferred to PVDF membrane for 2 h and then blots were blocked and incubated with PRSS3 antibody (Santa Cruz Biotechnology Inc., Santa Cruz, CA, USA) overnight at 4°C. Blots were washed and incubated with secondary antibody and detected with luminol chemiluminiscence reagent (Santa Cruz Biotechnology Inc.).

### Microarray

RNA from vaginal tissues from age-matched WT and *Fbln5^−/−^* mice (2 animals in each group) were subjected to microarray experiments. To control for influence of the hormonal environment, all animals were ovariectomized and treated with estrogen (E2) (50 µg/kg/d) ×3 d two weeks after ovariectomy. Affymetrix mouse expression chip 430 2.0 data was exported to the gene analysis software. Per chip normalization to the 50th percentile expression level and per gene normalization to the median expression of all samples was performed. Probe sets scored as either present or marginal were used in the data analysis, and average expression readings <100 in both WT and KO were excluded. Fold change expression was calculated as the median expression of WT over KO. Annotations for the probe sets were derived from the Affymetrix web site (www.affymetrix.com) and were updated with a variety of publicly accessible databases (National Center for Biotechnology Information [NCBI], Information Hyperlinked over Proteins [IHOP] and SwissProt database to verify gene identity and update annotations from current literature references. The microarray data from this publication have been submitted to the GEO database (http://www.ncbi.nlm.nih.gov/geo/) and assigned the accession number GSE40249.

### Statistical Analysis and Power Calculation

Statistical comparisons between groups were conducted by Student’s t test or, for multiple groups, one-way ANOVA using Dunnet’s comparison of means were used. P values ≤0.05 were considered significant. Based on the variability determined from pilot studies, we used a 2∶1 disease: control ratio to estimate a sample size of 8 controls and 16 prolapse to detect a 2-fold change in mRNA levels with a power of 0.80 and an alpha of 0.05. Since it was anticipated that up to 30% of RNA samples would be degraded or lost in sample preparation, 12–15 controls were used. For menopausal women with a wider age span and different stages and thereby increased variability, all apical samples available from the tissue bank were analyzed. Three samples were excluded due to incomplete clinical data. Data were reported as mean ± SEM.

## Results

### Caseinolytic Proteases in the Vaginal Wall of *Fbln5^−/−^* Mice

**Figure 1:**
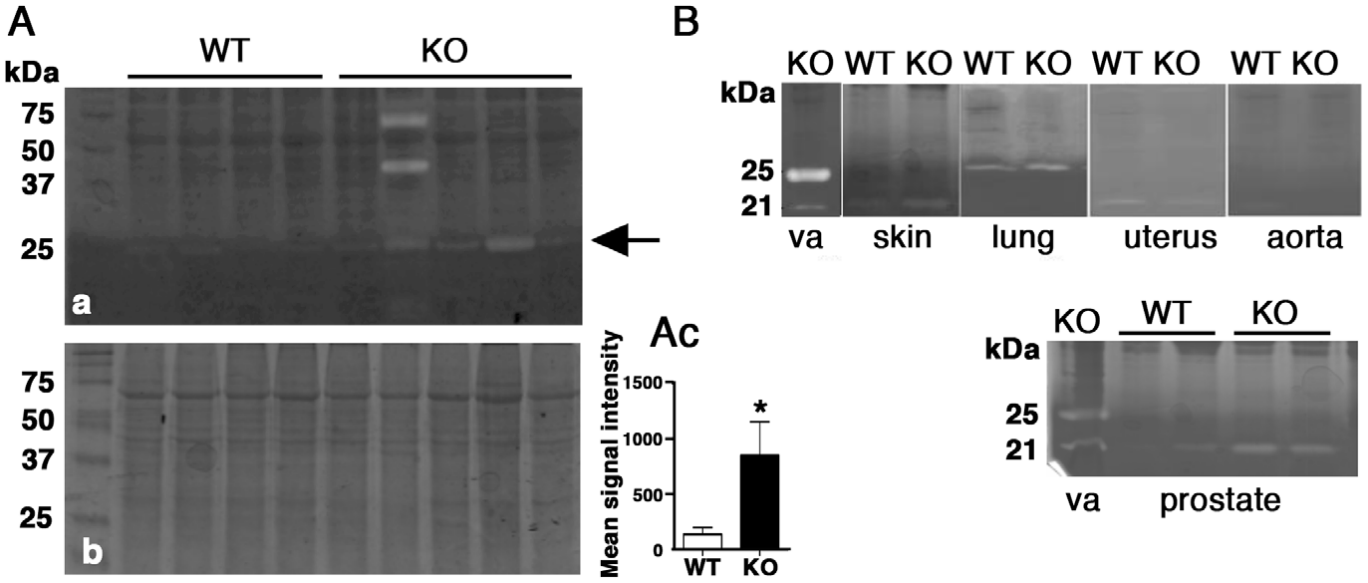
Detection of caseinolytic activity in *Fbln5^−/−^* tissues. (**A**) *a,* casein zymography using vaginal extracts from wild-type (WT) and *Fbln5^−/−^* (KO) at 1–2 months of age. Arrow indicates caseinolytic activity at 25-kDa. *b,* corresponding coomassie staining showing equal loading. *c*, Quantification of caseinolytic activity at 25-kDa in a. Bars are mean ± SEM. *P<0.05. (**B**) Caseinolytic activity was examined in various tissues from WT and KO mice. Note a 21-kDa band detected in vagina, skin, uterus, and prostate.

To search for protease(s) potentially involved in development and/or progression of POP, casein zymography was performed using vaginal tissues of WT and *Fbln5^−/−^* mice. Caseinolytic protease activity at 25 kDa was increased in vaginal tissues from *Fbln5^−/−^* mice (Figs. 1Aa and Ac, asterisk P = 0.0317) and was named V1. In some extracts, caseinolytic activity was observed at 21 kDa (Fig. 1B). To determine if these proteases were unique to the vaginal wall, casein zymography was conducted with extracts from vagina, prostate, skin, lung, uterus and aorta from WT and *Fbln5^−/−^* animals. Although differentially expressed in the vagina of *Fbln5^−/−^* mice, differences in the 25 kDa protease were not observed in the lung, and other tissues did not express the 25 kDa protease (Fig. 1B). It is of note that the 21 kDa caseinolytic protease was observed in skin, uterus and prostate of both WT and *Fbln5^−/−^* mice and upregulation was observed in the skin and prostate of *Fbln5^−/−^* mice.

**Figure 2:**
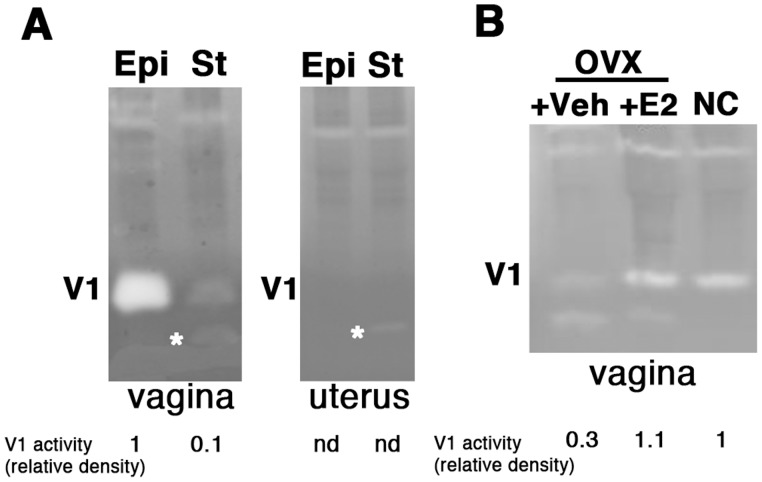
Compartmentalization of caseinolytic activity in *Fbln5^−/−^* vagina. (**A**) Epithelium (Epi)- and stroma (St)-enriched extracts were prepared from vagina (left) and uterus (right) from *Fbln5^−/−^* mice. Note strong V1 activity predominantly detected in epithelium of the vagina. An asterisk shows the activity at 21 kDa. V1 activity is indicated by relative density of the band. nd, not detected. (**B**) Effect of estrogen on V1 activity in *Fbln5^−/−^* mice. Extracts were prepared from vagina of normal cycling (NC) or ovariectomized animals injected with vehicle (+Veh) or estrogen (+E2).

To localize V1 in the vaginal wall, vaginal epithelium was scraped from the underlying stromal tissue of *Fbln5^−/−^* mice and both compartments subjected to casein zymography (Fig. 2A, vagina). V1 protease was highly enriched in epithelium compared to stroma in the vagina and it was not detected in the myometrial layer of the uterus (Fig. 2A, uterus). In both vagina and uterus, a faint band at 21 kDa was detected (Fig. 2A, asterisks). Since it is known that estrogen regulates the properties of vaginal epithelium, casein zymography was conducted in vaginal protein extracts from ovariectomized *Fbln5^−/−^* treated with or without estradiol and compared with that in normal cycling *Fbln5^−/−^* animals (Fig. 2B). V1 protease activity was decreased in vaginal tissues from ovariectomized mice compared with control (NC; normal cycling). Estradiol treatment restored V1 protease activity to normal levels. These results suggest that estrogen regulates the relative amounts of caseinolytic activities in the vaginal wall and uterus of knockout animals, and that the timing of induction and duration of expression may be regulated by estrogen.

### Characterization of V1 Protease

Although physiologic vaginal pH is maintained within the acidic range, various conditions such as menopause and infection may alter the pH. Thus, the effect of pH on caseinolytic activities was examined. Vaginal extracts from *Fbln5^−/−^* mice were subjected to casein zymography at various pH. V1 exhibited optimal activity at pH 8, with loss of activity at acidic or highly alkaline pH (Supporting Fig. S1).

**Figure 3:**
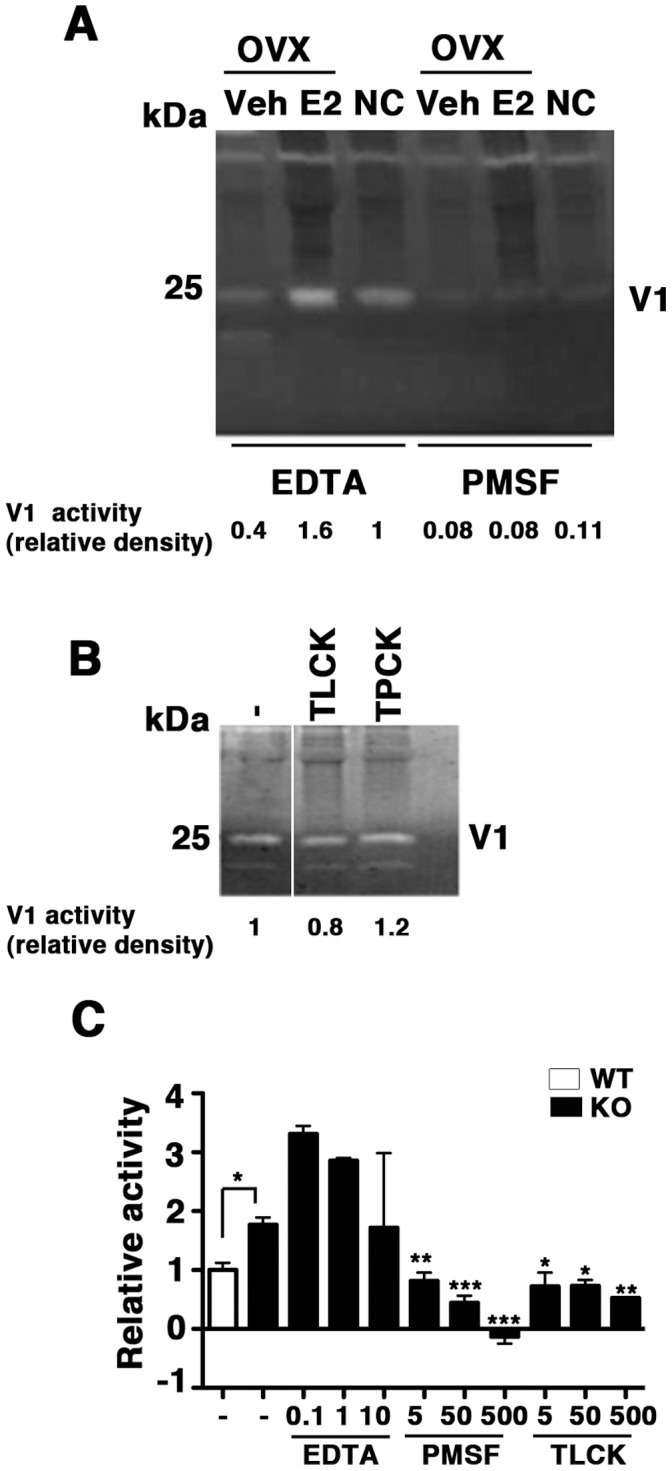
Effects of protease inhibitors on V1 activity. (**A**) Normal cycling and ovariectomized vagina extracts shown in Figure 2 were subjected to protease inhibition. EDTA (10 mM) or PMSF (100 µM) was incubated with each extract before casein zymography. Whereas EDTA showed no effect, PMSF completely inhibited V1 activity. V1 activity is indicated by relative density of the band. (**B**) *Fbln5^−/−^* vaginal extracts were pre-incubated with vehicle, TLCK (5 µM), or TPCK (10 µM) for 37°C for 1 h before analyzed by casein zymography. Note that V1 activity was resistant to protease inhibitors. (**C**) Caseinolytic activity assays using EDTA (0.1–10 mM), PMSF (5–500 µM), and TLCK (5–500 µM) were incubated with *Fbln5^−/−^* vaginal extracts and fluorescence intensity was measured as a caseinolytic activity. Note inhibition by PMSF and TLCK. Bars are mean ± SEM. *P<0.05, **P<0.01, and ***P<0.005 compared with KO without inhibitor.

Next, the protease inhibitor profile was determined with or without estrogen treatment. EDTA, a well-established inhibitor of MMPs, did not alter V1 activity. PMSF, a broad-spectrum serine protease inhibitor, however, inhibited V1 significantly (Fig. 3A). Under the present experimental conditions, V1 protease activity was inhibited by TLCK (specific for trypsin) only by 20% and resistant to TPCK (specific for chymotrypsin-like activity) (Fig. 3B). Consistent with these findings, caseinolytic activity assays using fluorescent substrates showed that the activity was resistant to EDTA and inhibited completely by PMSF (Fig. 3C). TLCK also inhibited the caseinolytic activity (Fig. 3C). The inhibitory effect of TLCK was not effective at a lower dose (Supporting Fig. S2A). Leupeptin (a competitive transition state inhibitor of serine, cysteine and threonine proteases such as trypsin, plasmin, kalliikrein, and cysteine proteases) and TPCK did not inhibit the caseinolytic activity (Supporting Fig. S2A). Pepstatin A (an inhibitor of aspartic proteases such as pepsin and cathepsins), and E64 (an irreversible, potent, and highly selective cysteine protease inhibitor) showed the inhibitory effects on the caseinolytic activity (Supporting Fig. S2B), although the inhibition was much weaker than that of PMSF (Fig. 3C).

**Figure 4:**
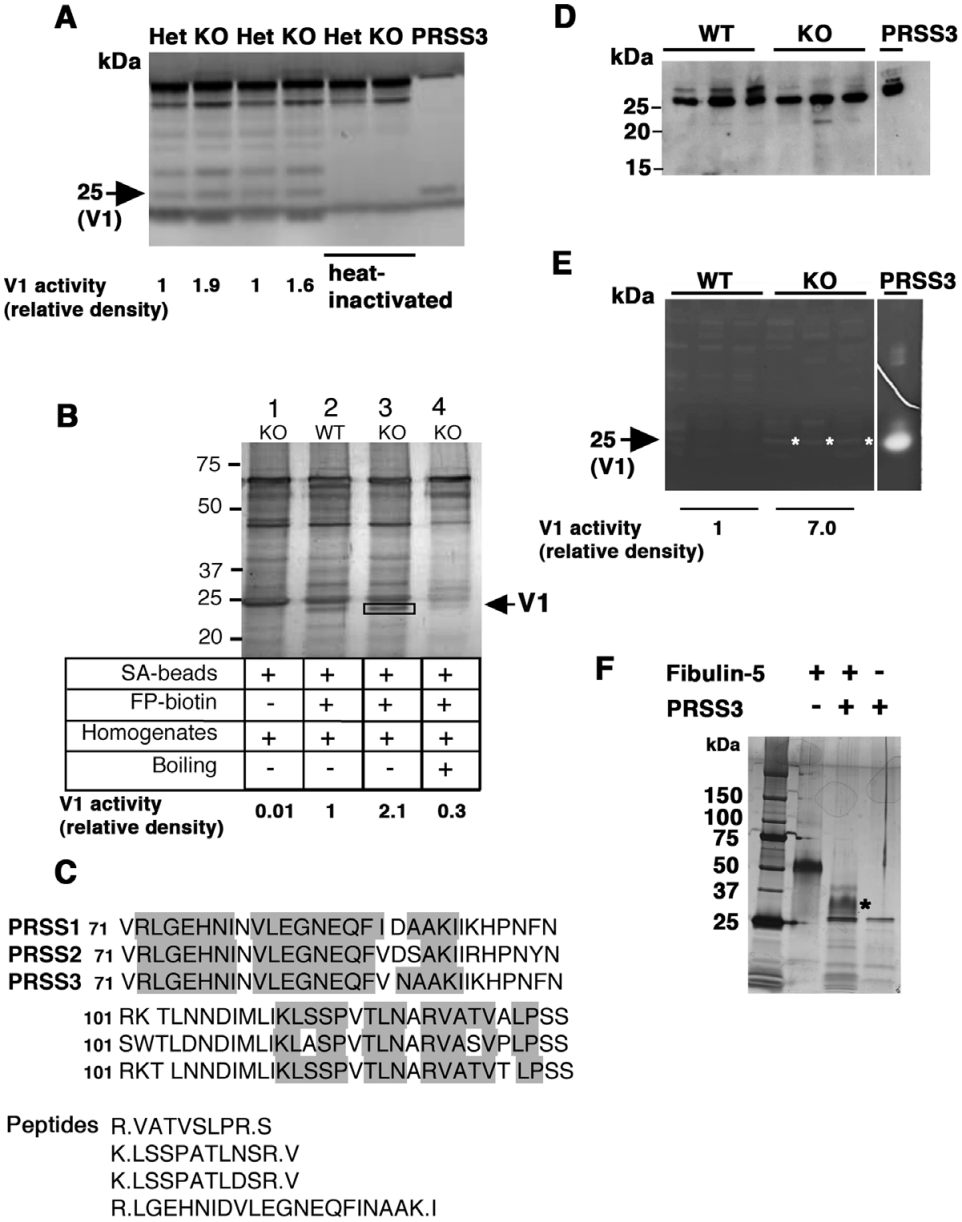
V1 shares similar biochemical properties as those of PRSS3. (**A**) Activity-based detection of serine proteases in vaginal extracts using TAMRA FP probe. PRSS3 recombinant protein was used as positive control for detecting 25 kDa serine protease activity. A band corresponding to 25 kDa along with other bands were seen in both *Fbln5^+/−^* (Het) and KO vaginal extracts. V1 activity is indicated by relative density of the band. (**B**) Activity-based pull-down assay of V1 protease. Up-regulation of ∼25-kDa FP-biotin reactive band is seen in the KO vagina (lane 3, box) compared to WT (lane 2). No corresponding band was seen in lane 1, in which KO extract was incubated with streptavidin (SA) beads without FP-biotin (lane 1). V1 activity is indicated by relative density of the band. (**C**) Alignment of PRSS1-3 proteins with peptide fragments (shaded box) obtained from 25-kDa band (box in B) after mass spectrometery. (**D**) Immunoblot analysis of PRSS3 using conditioned media of vaginal organ culture from WT and KO mice. Note comparable levels of secreted immunoreactive PRSS3 in WT and KO. (**E**) Casein zymography using conditioned media described in (D). PRSS3 was used as positive control to show the caseinolytic activity of the recombinant enzyme. Note caseinolytic activity at 25 kDa (asterisk) was increased in KO conditioned media relative to that in WT. V1 activity was indicated by relative density of 25 kDa band (average of three samples). (**F**) In vitro cleavage of fibulin-5 by PRSS3. Note asterisk indicates a major cleavage product of fibulin-5. The experiment was performed three times.

To confirm that V1 was a serine protease and provide a better estimate of its molecular size, a TAMRA-fluorophosphonate (FP) probe was used to conduct activity-based protein profiling of vaginal tissues from *Fbln5* heterozygous (Het) and knockout (KO) mice (Fig. 4A). FP is an irreversible inhibitor of fluorophosphonate/fluorophosphate derivatives and preferentially inhibits serine proteases [12]. The reactivity of FP requires the presence of a catalytically active serine hydrolase. Multiple proteases were labeled by FP indicating multiple catalytically active serine proteases in the vaginal wall. Most labeled proteases, however, were similar in KO and Het animals. In contrast, V1 protease was increased in vaginal extracts from KO animals. Based on these results, we performed activity-based pull down assays of V1 protease using a biotinylated FP probe [12] (Fig. 4B). Consistent with TAMRA-FP probe, upregulation of a ∼25-kDa FP-reactive band was detected in KO extracts, which was abolished by heat inactivation. The band was excised and sequenced by mass spectrometry. Four sequences were obtained that partially matched to PRSS1-3 (Fig. 4C). Taken together, the biochemistry, inhibitory profile, activity-based protein profiling, and mass spectrometry data suggest that V1 protease is a trypsin-like, but not chymotrypsin-like, serine protease similar to PRSS1-3 trypsinogens.

### Candidates for Increased Caseinolytic Proteases in the KO Vagina

**Figure 5:**
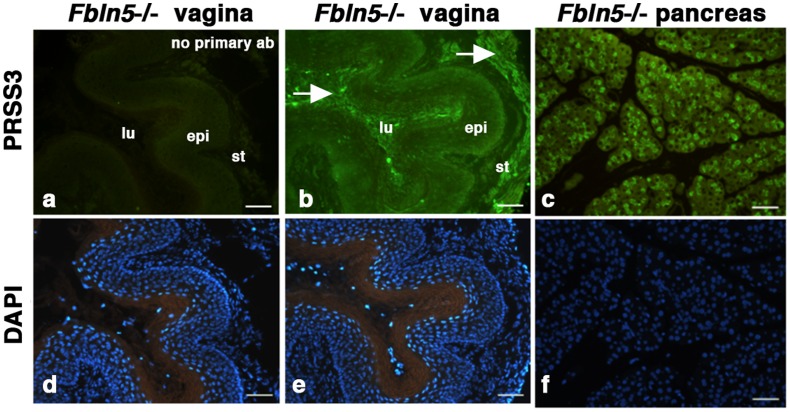
PRSS3 immunoreactivity in *Fbln5^−/−^* vagina. Vaginal (a, b, d, e) or pancreas sections (c, f) from 1-month old *Fbln5^−/−^* mouse were incubated with secondary antibody only (a) or anti-PRSS3 antibody (b, c) and counter-stained with DAPI (d, e, f). Note strong PRSS3 expression in vaginal secretions as well as in stroma of the *Fbln5^−/−^* vagina. lu, vaginal lumen; epi, epithelium; st, stroma. Bars are 30 µm.

Based on its (i) molecular size, (ii) mass spectrometric analysis, (ii) resistance to common trypsin inhibitors and iii) optimal pH, we concluded that V1 is most likely PRSS3. PRSS3, together with PRSS1 and PRSS2, comprise a trypsinogen family. While PRSS1 and PRSS2 are major pancreatic trypsinogens, PRSS3 is shown to be expressed both in the pancreas and extra-pancreatic tissues, including the brain and skin [13], [14]. To examine if PRSS3 is secreted from vaginal tissues, vagina organ culture was prepared from wild-type and *Fbln5^−/−^* mice and conditioned media were collected. Western blot analysis of conditioned media showed cross-reactivity to PRSS3 at approximately 25 kDa both in wild-type and knockout mice (Fig. 4D). PRSS3 levels, however, did not differ. In contrast, 25-kDa caseinolytic activity was increased in conditioned media from *Fbln5^−/−^* vagina relative to almost non-detectable activity in wild-type (Fig. 4E), suggesting the possibility that the PRSS3-like enzyme activity is primarily regulated by protease inhibitors. We then asked if PRSS3 could cleave fibulin-5. Fibulin-5 is shown to be cleaved at arginine 77 and the cleaved form of fibulin-5 is unable to assemble elastic fibers in elastogenic assays [15]. Incubation of fibulin-5 with PRSS3 yielded cleaved products with a major band at approximately 30-kDa (asterisk in Fig. 4F), demonstrating that PRSS3 was able to cleave fibulin-5 in vitro. Immunostaining with PRSS3 antibody confirmed immunoreactivity was present in vaginal secretions in lumen and stroma of *Fbln5^−/−^* vagina (arrows in Fig. 5). The specificity of the immunostaining was confirmed by directly treating the vaginal sections with secondary antibody and by omitting primary antibody from the procedure. These results suggest that V1 most likely represents vaginal PRSS3-like enzyme but V1 activity may be influenced by inhibitor levels in the vaginal tissues and that the balance between protease and inhibitors may be altered in the *Fbln5^−/−^* vagina. Furthermore, PRSS3 may cleave fibulin-5 and regulates fibulin-5 levels in the vaginal wall.

### Potential Regulation of Serine Protease Inhibitors in the Vaginal Wall

**Figure 6:**
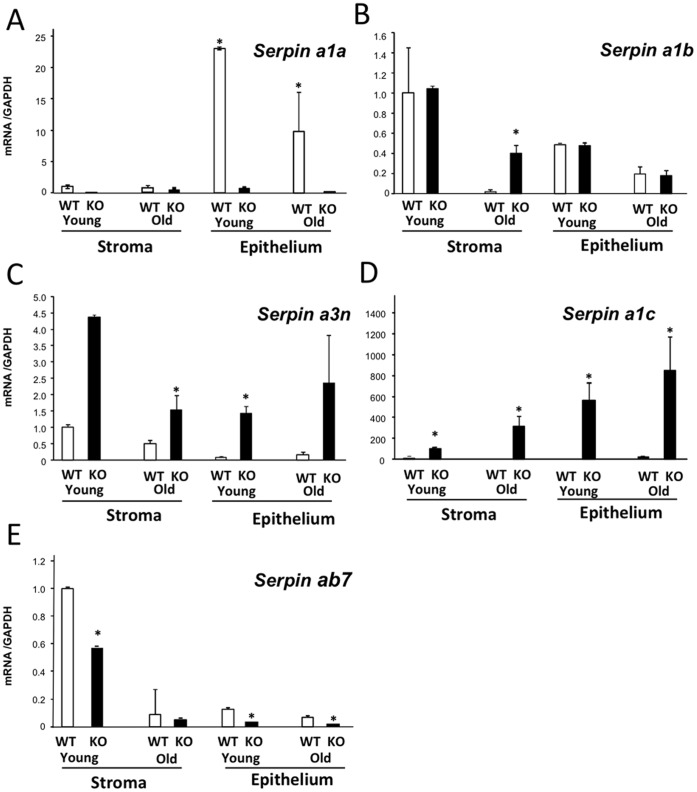
Tissue-specific expression of select Serpins in vaginal stroma and epithelium from young (4–6 wks) and old (6–8 mo) WT and *Fbln5* ^−/−^ (KO) mice. Epithelium was scraped from the underlying stroma of the vaginal wall from WT and KO mice. Expression of *Serpins a1a* (**A**), *a1b* (**B**), *a3n* (**C**), *a1c* (**D**), and *ab7* (**E**) was analyzed by qPCR. Epithelium from 3–4 mice was pooled for one data point. Data represent mean ± SEM of epithelium (n = 3 pools) or stroma (n = 8−10) from each genotype at each age. *P<0.05 compared with corresponding WT.

Our findings suggesting that vaginal PRSS3 activity is primarily regulated by its inhibitors led us to investigate serine protease inhibitors in the vaginal wall. Using microarray analysis, we were surprised to find striking upregulation of certain protease inhibitors in the vaginal wall from *Fbln5^−/−^* mice (Table II). Specifically, a number of highly homologous serpin family members were differentially expressed in the vaginal wall of *Fbln5^−/−^* mice, including *Serpina1a*, and *Serpina1b* (α1-antitrypsin and its z variant), *Serpina1c* (Elafin, also termed SKALP), and *Serpina3n* (α1 antichymotrypsin). To differentiate these protease inhibitors, we quantified relative mRNA levels in vaginal tissues as a function of age in epithelium and stroma from WT and *Fbln5^−/−^* mice (Fig. 6). The serine protease inhibitor, *Serpina1a*, was predominantly expressed in the epithelium of WT mice. Expression of *Serpina1a*, but not *Serpina1b*, was significantly decreased in vaginal epithelium from both young and old *Fbln5^−/−^* mice (Figs. 6A,B). In contrast, *Serpina3n* was increased ∼4-fold in both stroma and epithelium from *Fbln5^−/−^* mice (Fig. 6C). The most striking changes were found with *Serpina1c* (Elafin) (Fig. 6D). *Serpina1c* was either not expressed or expressed at very low levels in the vaginal wall from WT mice. In contrast, its expression was increased dramatically in vaginal tissues from *Fbln5^−/−^* mice (i.e., 600- to 800-fold in vaginal epithelium from young and old *Fbln5^−/−^* animals). Consistent with microarray results, *Serpinb7* (Megsin) was decreased in both stroma and epithelium of *Fbln5^−/−^* mice (Fig. 6E). Overall, these data indicate that loss of *Serpina1a* and *Serpinb7* in the epithelium of *Fbln5^−/−^* mice may contribute to increased serine protease activity in the epithelium of these animals. Furthermore, the striking upregulation of *Serpina1c* in epithelium of *Fbln5^−/−^* animals may represent a compensatory gene regulatory event to abbreviate or suppress excessive protease activity in the vagina. Alternatively, fibulin-5 or fibulin-5-mediated fully assembled elastic fibers, localized in the matrix of vaginal stroma [9] may signal epithelial cells to suppress *Elafin* gene expression.

**Table 2:** Protein inhibitors expressed in the vaginal wall as determined using microarray analysis of vaginal tissues from estrogen-treated wild type and *Fbln5^−/−^* mice.

Unigene or Genbank ID	Gene	Subcellular localization/size	Fold-change*
NM_009243	Serpina1a	Secreted/413	19.2
NM_138684	Elafin-like protein	Secreted/71	9.2
NM_009245	Serpina1c	Secreted/413	6.3
BC012874.1	Serpina1b	Secreted/413	4.9
NM_009252.1	Serpina3n	Secreted/418	4.3
Mm.83909	Serpinb8	Intracellular/373	no change
Mm.123919	Serpinb10	Intracellular/396	no change
Mm.66015	Serpinb7	Intracellular/379	−3.3
Mm.2044	Serpinf1	Secreted/416	−5.3

* Fold change in *Fbln5^−/−^* vagina by microarray.

### Serine Protease PRSS3 in Vaginal Tissues from Women with and without POP

**Figure 7:**
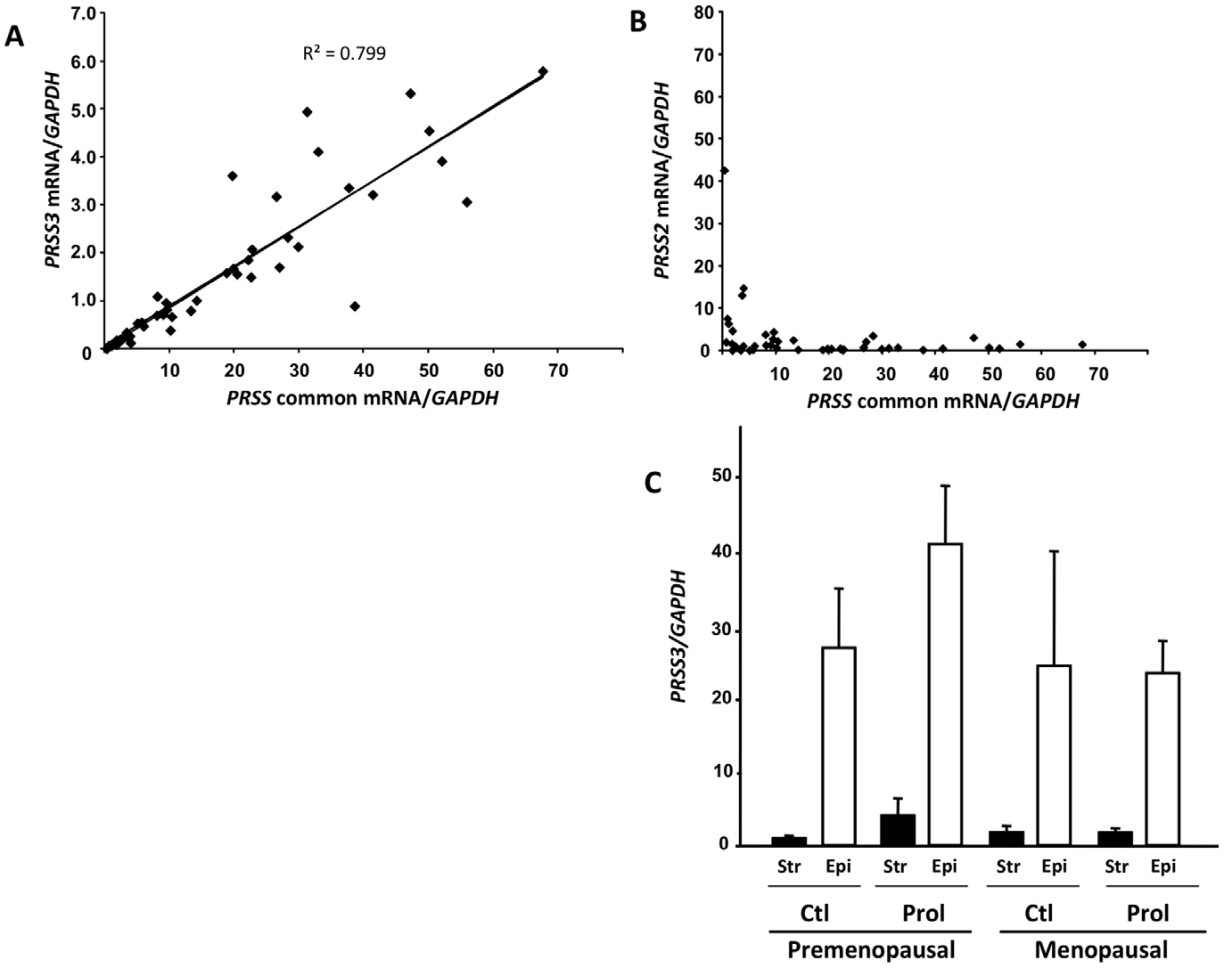
Expression of *PRSS3* mRNA in the human vagina. Expression of *PRSS* quantified by qPCR using primers that recognize all 3 family members (PRSS common) correlated with expression of primer-specific *PRSS3* (**A**), but not *PRSS2* (**B**). **C**. *PRSS3* mRNA levels in vaginal stroma (**Str, solid bars**) or epithelium (**Epi, open bars**) from pre- or post-menopausal women with (**Prol**) or without (**Ctl**) pelvic organ prolapse. Data represent mean ± SEM of stroma samples described in Table 1. For epithelium: pre ctl, n = 5; pre prol, n = 8, post ctl, n = 3; post prol, n = 12.

Our data in mice indicated PRSS3 as a potential candidate for excessive caseinolytic protease activity in the vagina from *Fbln5^−/−^* mice, and that dysregulation of protease inhibitors may effect these changes. To further substantiate the role of these proteases and their inhibitors in the context of human POP, similar studies were carried out using vaginal muscularis and mucosa (i.e., epithelium) from women with and without POP. First, we tested the hypothesis that PRSS family members were expressed in the vaginal wall of humans using primers that recognize all 3 family members (Prss1, 2, and 3). Since PRSS was highly expressed in vaginal epithelium (i.e., C_T_ = 22−23 using 25 ng RNA), but rarely in vaginal stroma, member-specific primers were used to identify PRSS3 as the sole PRSS protease expressed in the vagina (Fig. 7A), except one menopausal subject with Stage 4 prolapse in which PRSS2 was identified (Fig. 7B). Nonetheless, *PRSS3* mRNA levels were not expressed differentially between controls and women with prolapse (Fig. 7C).

### Serine and MMP Protease Inhibitors in the Human Vaginal Wall

**Figure 8:**
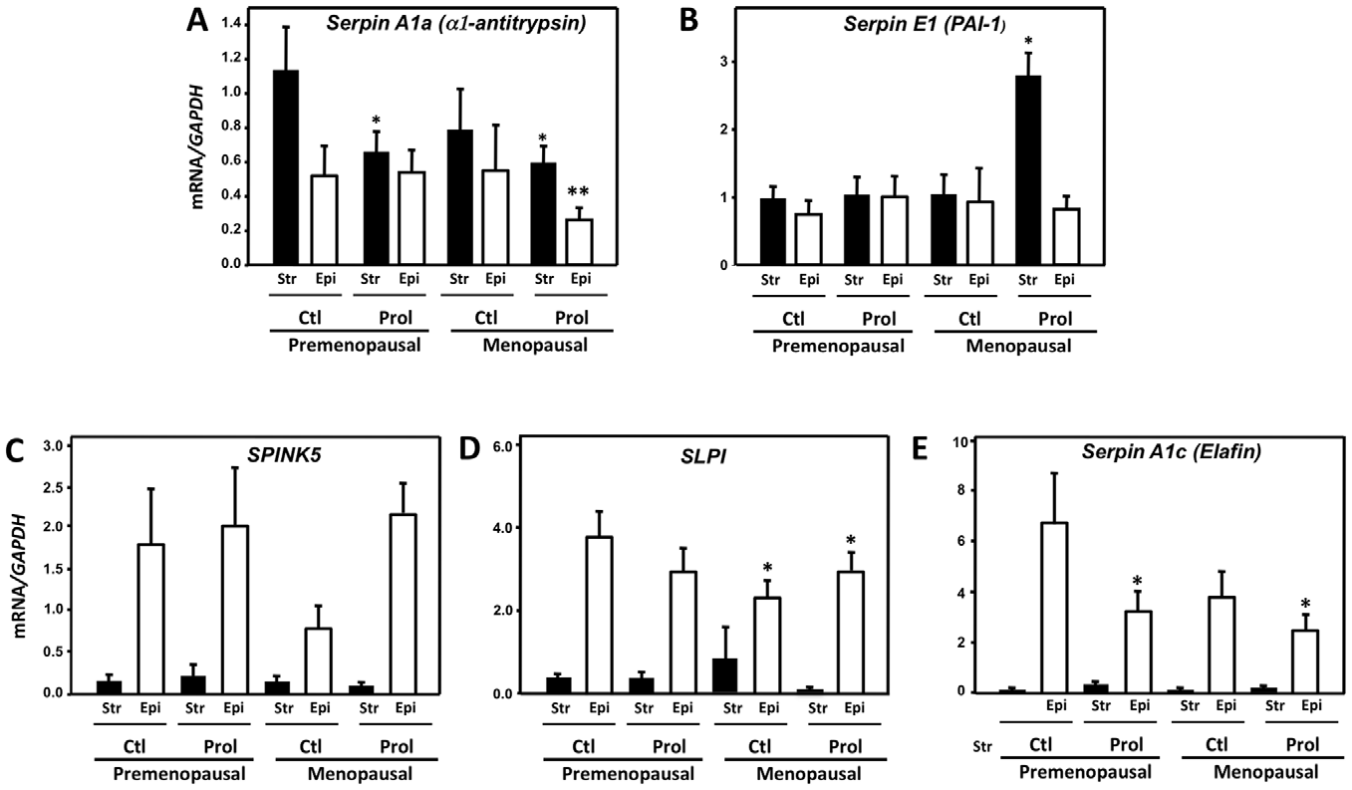
Tissue-specific expression of serine protease inhibitors in the vaginal wall. *SERPINA1* (**A**) and *E1* (**B**) were expressed in vaginal stroma (**Str, solid bars**) and epithelium (**Epi, open bars**) from pre- or post-menopausal women with (**Prol**) or without (**Ctl**) pelvic organ prolapse. Serine protease inhibitors *Spink5* (**C**), *SLPI* (**D**), and *Elafin* (**E**) were predominantly expressed in vaginal epithelium. Data represent mean ± SEM of stroma samples described in Table 1. For epithelium: pre ctl, n = 5; pre prol, n = 8, post ctl, n = 3; post prol, n = 12. *P<0.05 compared with stroma from premenopausal controls, ANOVA, Dunnett’s post hoc testing. **P<0.01.

To investigate the possibility that serine protease inhibitors may be altered in the prolapsed vaginal wall, expression of *SERPINA1* and *E1* (α1-antitrypsin and plasminogen activator inhibitor-1, respectively) was quantified in vaginal tissues from pre- and post-menopausal women with and without POP. Unlike mice in which *Serpina1a* was limited to the epithelium, expression of *SERPINA1* was expressed in both stromal and epithelial compartments of the human vaginal wall (Fig. 8A). Compared with stroma from control premenopausal women, *SERPINA1* mRNA was decreased modestly in stromal tissues from pre- and postmenopausal women with prolapse. Furthermore, *SERPINA1* was also decreased significantly in epithelium from menopausal women with prolapse. *SERPINE1*, on the other hand, was increased significantly in stroma from menopausal women with prolapse (Fig. 8B). The distribution pattern of serine protease inhibitor kazal-types 5 (*SPINK5*), secretory leukocyte protease inhibitor (*SLPI*), and *PI3* (*Elafin*) were distinct compared with *SERPINA1* and *SERPINE1* (Figs. 8C,D). All were highly expressed in epithelium, not stroma. Although *SPINK5* and *SLPI* expression was not changed with prolapse or menopausal status, *PI3* (*Elafin*) was decreased significantly in both pre- and post-menopausal women with prolapse compared with premenopausal controls (Fig. 8E), unlike increased expression of *Elafin* (*Serpina1c*) found in *Fbln5^−/−^* mice. These data suggest that loss of Elafin may result in increased secretion of bioactive serine proteases in the vaginal wall in women.

Previously, we demonstrated increased MMP-9 activity in vaginal stroma from women with POP [9]. To add insight into the potential mechanisms of this upregulation, we quantified relative mRNA levels of tissue inhibitor of metalloprotease (*TIMP)1* and *TIMP2* in vaginal tissues from women with and without prolapse. Expression of *TIMP2* was decreased in epithelium of menopausal women regardless of prolapse status (P<0.05) suggesting that estrogen deficiency may lead to loss of MMP inhibitors in the epithelial layer (Supporting Fig. S3).

## Discussion

POP is a multifactorial disease involving structural changes of vaginal wall. Our recent study has shown that defective assembly of elastic fibers and loss of fibulin-5-mediated inhibition of MMP-9 lead to weakening of the vaginal wall by facilitating secondary degradation of collagen fibers. In this study, we extended our understanding of the mechanisms underlying POP to include dysregulation of an important serine protease that catalyzes degradation of fibulin-5 in vitro.

### V1 is a 25-kDa PRSS3-like Serine Protease in the Vaginal Wall

Several studies have shown secreted protease activities in the vagina, but our studies indicate that V1 is unique. For example, caseinolytic activity of 26-kDa was previously reported in cycling mouse vagina but this protease was dramatically upregulated after ovariectomy and was not gelatinolytic, suggesting that this was a plasmin-like enzyme. In humans, KLKs 6, 7, 8. 10, 11, 12 and 13 have been found in cervico-vaginal fluid with peak levels after ovulation at around day 20 of the menstrual cycle [16]. In contrast, trypsin-like activity in cervico-vaginal fluid peaked around the time of ovulation in human samples [16]. Trypsin-like activity has also been reported in rat vaginal epithelial cells and the activity peaked during proesterus, when plasma estrogen was maximal, and its activity was optimal around pH 7 [17]. Our data indicate that caseinolytic protease activity (termed V1) is also upregulated by estrogen and predominantly secreted from epithelium in *Fbln5^−/−^* vagina, suggesting that V1 is similar to trypsin-like enzyme previously reported from vaginal epithelium. Although it is currently undetermined whether V1 protease is molecularly identical to PRSS3 or any isoform of PRSS3, V1 shares similar biochemical characteristics to PRSS3 because (i) the similar molecular size, (ii) it functions at a neutral pH, (iii) is resistant to several serine and cysteine protease inhibitors, EDTA, and common trypsin inhibitors, and (iv) immunoreactive PRSS3 is secreted from vaginal tissues. We confirmed that both PRSS3 and V1 contain caseinolytic and gelatinolytic activity at 25 kDa. PRSS3 represents <10% of total trypsinogen in normal pancreatic juice (reviewed in [18]). PRSS3 is (i) resistant to SBTI, PSTI, and BPTI (ii) sensitive to PMSF and TLCK [19], and (iii) requires proteolytic activation by enterokinase [19]. A new alternative form of PRSS3 was reported in differentiated cultured keratinocytes and was named trypsinogen 5 [13]. Unlike the relatively broader distribution of another alternative isoform (i.e., trypsinogen 4), trypsinogen 5 was restricted to the brain, small intestine, uterus and keratinocytes. Both trypsinogen 4 and 5 differ only in the N-terminus and both produce active PRSS3 upon cleavage by enteropeptidase, which is shown to be expressed in the granular layer of the epidermis. Therefore, V1 may undergo proteolytic activation by other enzymes in vaginal epithelial cells.

### Mechanism of Upregulation of V1 Protease in Vaginal Wall

The process of enteropeptidase activation is important in regulation of PRSS protease activity both in the pancreas and in cancer cells in which addition of enteropeptidase enhanced cell-mediated proteolysis [20]. Currently, the molecular mechanisms of V1 activation are not known. In addition to enteropeptidase, MMP-9 is also known to activate trypsinogen family members in the pancreas [21]. Previously, we demonstrated that fibulin-5 inhibits MMP-9 in a RGD-dependent manner and that MMP-9 is thereby upregulated in the *Fbln5^−/−^* vagina. It is tempting to speculate, therefore, that the elevated levels of vaginal MMP-9 in the absence of fibulin-5 may lead to activation of V1. Alternatively, V1 may be tethered to elastic fibers in the vaginal wall as a pro form. Destruction of elastic fibers itself may release V1 and increase bioavailability of these enzymes. Currently, the mechanisms of V1 enzyme activation are simply unknown but under active investigation.

### Potential role of V1 Protease Activity

Proteases play a crucial role in extracellular matrix remodeling through their proteolytic action on collagens, proteoglycans, fibronectin, elastin and laminin. The vaginal epithelium represents a major interface between the external environment and the female reproductive tract. It is constantly exposed to proteolytic enzymes from many sources, including bacteria in the vaginal vault and immune cells in the lamina propria and subepithelium. Controlled proteolytic activity is thereby important for maintainance of normal tissue turnover and maintenance of this barrier. It is plausible that the V1 proteolytic activity may contribute to the pathophysiology of POP. A similar trypsinogen secreted from cancer cell lines, degrades subendothelial cell extracellular matrix (ECM) and it has been shown that enzymes similar to PRSS3 degrade fibronectin and aggrecan [20], [22]. Recently, another serine protease termed HTRA1 (high-temperature requirement factor A1, previously named PRSS11) with a highly conserved trypsin-like protease domain similar to PRSS3 was shown to alter Bruch’s Membrane composition in vivo and rHTRA1 lacking the N-terminal domain cleaved various extracellular matrix (ECM) proteins, including fibronectin and fibulin-5 [22]. Here, we showed that PRSS3 also cleaved fibulin-5 in vitro. Thus, dysregulated V1 activity during the elastogenesis period may result in excessive cleavage of fibulin-5 and lead to disorganized elastic fibers in the vaginal wall. Since we have previously shown in *Fbln5^−/−^* vagina that elastic fibers connecting epidermis and stroma were loose and disrupted, it is fascinating to speculate that uninhibited trypsin-like proteases such as V1/PRSS3 are involved in destruction of the integrity of connective tissues in subepidermis by cleaving fibulin-5 and other elastogenesis-associated ECM. Although loss of fibulin-5 after the completion of elastogenesis may not influence resting adult tissues, the continuous remodeling of the female reproductive tract during reproductive life [23] which is accelerated after childbirth [24], and loss of fibulin-5-mediated inhibition of vaginal MMP-9 is predicted to have adverse effects on maintenance of vaginal connective tissues, especially postpartum when fibulin-5 protein levels return to baseline levels [1].

### Proteases and Proteinase Inhibitors in POP

Our findings showing that the imbalance between protease and protease inhibitors are associated with POP phenotype in mouse and human adds complexity to our understanding the pathogenesis of POP. We observed upregulation of MMP-9 as well as a trypsin-like serine protease in the *Fbln5^−/−^* vagina, and we confirmed that *PRSS3* was indeed expressed in human vaginal tissues. Interestingly, in the human vagina, *PRSS3* was also limited to the epithelium but mRNA levels were not increased in vaginal tissues from women with prolapse. Limitations in the amount of tissue available precluded a comprehensive analysis of 25 kDa enzyme activity in the human. Nonetheless, SERPINA1 (α1-antitrypsin) was decreased in stromal tissues from pre- and postmenopausal women with prolapse and in epithelium from menopausal women with POP. *Elafin* was also decreased significantly in tissues from women with prolapse. The role of α1-antitrypsin has been well studied in pulmonary emphysema, in which reduction of α1-antitrypin leads to destruction of elastin ECM, resulting in enlargement of alveoli (Reviewed in [25]). Conversely, overexpression of α1-antitrypsin in cigarette smoke-induced and VEGF-inhibition-induced, proteolysis-independent pulmonary emphysema models suggested that α1-antitrypsin serves not only as an inhibitor of elastase but also possesses elastase-independent anti-apoptosis functions in vivo [26], [27]. It has been suggested that apoptotic cells are increased in pelvic tissues from women with prolapse [28], and α1-antitrypsin mRNA was reported to be decreased in postmenopausal women with POP relative to postmenopausal women without POP [29]. Another report, however, did not find any positive correlation between the site of α1-antitrypsin expression and posterior/anterior ratio in bladder and uterine prolapse [30]. Therefore, our report has shown for the first time that α1-antitrypsin levels were decreased in menopausal women with POP compared to control.

It is interesting to note that in some cases, protease regulation by inhibitors differed in humans and *Fbln5^−/−^* mice with prolapse. For example, the serine protease inhibitor, Elafin, which is regulated primarily by its transcript level [31], was decreased in epithelium from women with prolapse. In contrast, we observed marked upregulation of Elafin transcripts in epithelium of *Fbln5^−/−^* vagina. In the complete absence of fibulin-5, Elafin may not localize to the matrix and its mRNA may be upregulated dramatically as a compensatory manner. In women with prolapse, however, in which fibulin-5 may be compromised [32]–[35] but not absent, Elafin may play a more substantial role in inhibiting elastase-mediated matrix degradation. Although *SERPINB7* was not investigated with adequate power, our pilot studies indicated no change in *SERPINB7* in vaginal stroma from women with or without prolapse (not shown). Further studies are necessary to provide a more in-depth understanding of each protease-protease inhibitor combination, how they affect overall protease activity, whether they play a causal role in progression of POP, and how, or if, fibulin-5 directly or indirectly regulates levels of protein inhibitor/protease complexes in the vaginal wall. Nevertheless, experimental data provided herein, together with previous investigations, indicate that proteases may regulate matrix integrity not only through direct degradation of collagen and elastin but also through other matrix components such as fibulin-5, a major MMP9 inhibitor in the vaginal wall.

## Supporting Information

Figure S1 **Effects of pH on V2 and V1 caseinolytic activities.** pH is indicated above each gel. Note that strongest activity was detected at pH 8.
(PDF)

Figure S2 **A. Caseinolytic activity assays with Leupeptin (0.1, 1, 10 µg), TLCK (0.1, 1, 10 µM), TPCK (0.1, 1, 10 µM) and SBTI (0.1, 1, 10 µg) using 4 µg of KO extracts.** **B.** Caseinolytic activity assays with Pepstatin A (0.1, 1, 10 µg) and E64 (0.5, 5, 50 µg) using 2 µg of KO extracts. Inhibitors were incubated with extracts and fluorescence intensity was measured as caseinolytic activity. Bars are mean ± SEM. KO activity without inhibitor was designated as 1.
(PDF)

Figure S3 **Tissue-specific expression of** ***TIMP1*** **and** ***TIMP2*** **in the vaginal wall.** *TIMP1* (**A**) and *TIMP2* (**B**) were expressed in vaginal stroma (**Str, solid bars**) and epithelium (**Epi, open bars**) from pre- or post-menopausal women with (**Prol**) or without (**Ctl**) pelvic organ prolapse. *P<0.05 compared with stroma from premenopausal controls, ANOVA, Dunnett’s post hoc testing.
(PDF)

Table S1 **Primers used for qPCR amplification.**
(DOCX)
