# Supplementary figures and images for: Dysregulation of Protease and Protease Inhibitors in a Mouse Model of Human Pelvic Organ Prolapse
Source: PLoS One. 2013 Feb 20;8(2):e56376. doi: 10.1371/journal.pone.0056376 (PMC3577807; doi:10.1371/journal.pone.0056376)

Figure S1

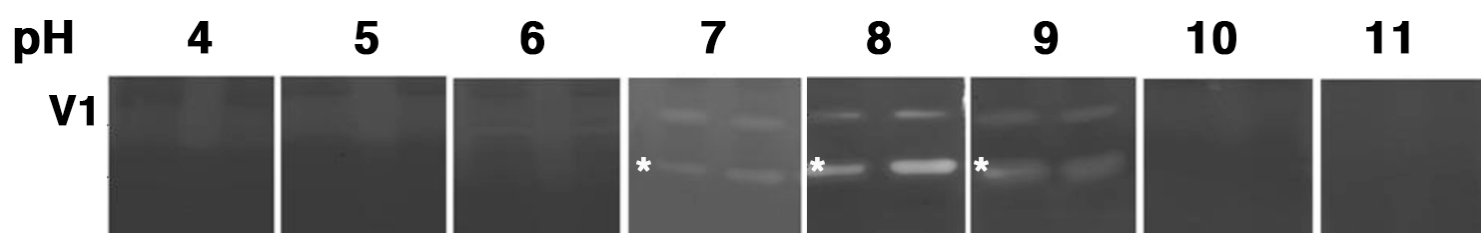

Supplement: Figure S1 — Effects of pH on V2 and V1 caseinolytic activities. pH is indicated above each gel. Note that strongest activity was detected at pH 8. (PDF) [file pone.0056376.s001.pdf]

Figure S2

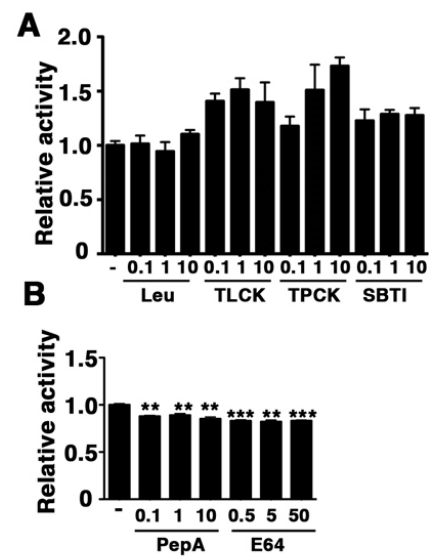

Supplement: Figure S2 — A. Caseinolytic activity assays with Leupeptin (0.1, 1, 10 µg), TLCK (0.1, 1, 10 µM), TPCK (0.1, 1, 10 µM) and SBTI (0.1, 1, 10 µg) using 4 µg of KO extracts. B. Caseinolytic activity assays with Pepstatin A (0.1, 1, 10 µg) and E64 (0.5, 5, 50 µg) using 2 µg of KO extracts. Inhibitors were incubated with extracts and fluorescence intensity was measured as caseinolytic activity. Bars are mean ± SEM. KO activity without inhibitor was designated as 1. (PDF) [file pone.0056376.s002.pdf]

Figure S3

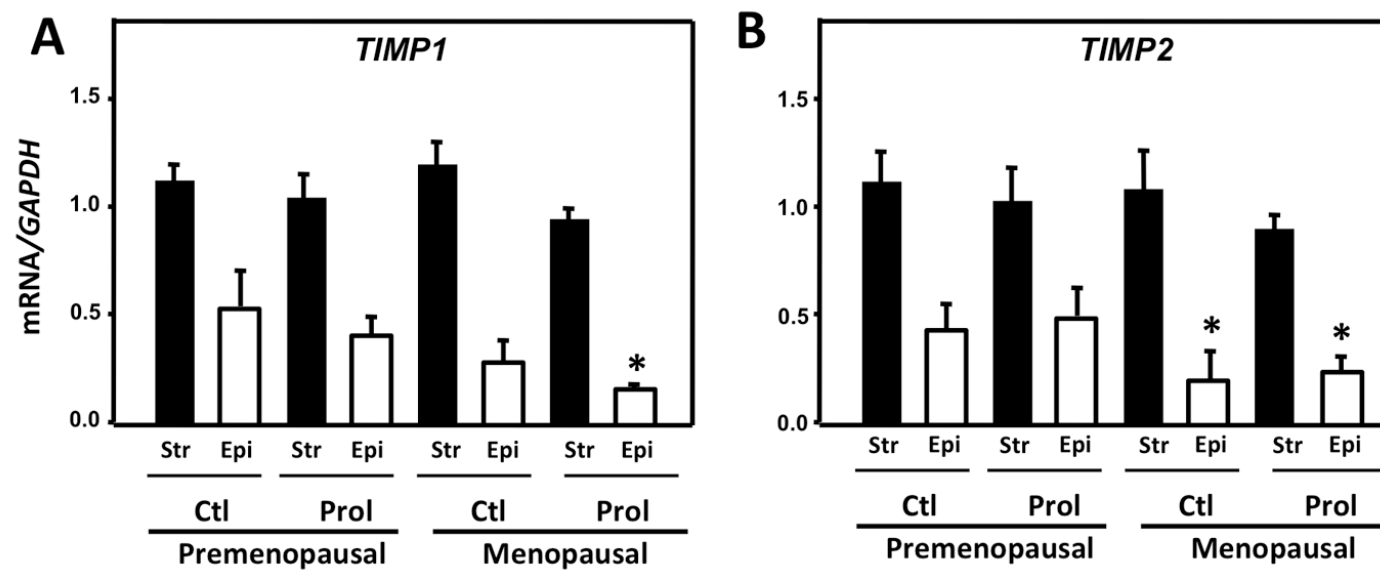

Supplement: Figure S3 — Tissue-specific expression of TIMP1 and TIMP2 in the vaginal wall. TIMP1 (A) and TIMP2 (B) were expressed in vaginal stroma (Str, solid bars) and epithelium (Epi, open bars) from pre- or post-menopausal women with (Prol) or without (Ctl) pelvic organ prolapse. *P<0.05 compared with stroma from premenopausal controls, ANOVA, Dunnett’s post hoc testing. (PDF) [file pone.0056376.s003.pdf]
