# Supplementary material for: Dysregulation of Protease and Protease Inhibitors in a Mouse Model of Human Pelvic Organ Prolapse
Source: PLoS One. 2013 Feb 20;8(2):e56376. doi: 10.1371/journal.pone.0056376 (PMC3577807; doi:10.1371/journal.pone.0056376)
Supplement: Table S1 — Primers used for qPCR amplification. (DOCX) [file pone.0056376.s004.docx]

**Table S1.**

| **Gene** | **Forward primer** | **Reverse primer** | **Accession #** |
| --- | --- | --- | --- |
| hPRSS common | CAGGCCTGGAGTCTACACCAA (678-698) | GGTGTCCTTAATCCAGTCCACATAG (732-708) | BC103998.2 |
| hPRSS3 | CACCCTAAATACAACAGGGACACT (299-322) | GACACGCGGGCATTGAT (384-368) | BC069476 |
| hPRSS 2 | GGAATGAACAGTTCATCAATGCA (254-276) | GTCCAGAGTCCGGCTGTTGT (321-302) | NM002770.2 |
| hTIMP1 | GACGGCCTTCTGCAATTCC (288-306) | GTATAAGGTGGTCTGGTTGACTTCTG (366-341) | NM003254.2 |
| hTIMP 2 | CCCTCCTCGGCAGTGTGT (556-573) | CGGCCTTTCCTGCAATGA (628-611) | BC052605 |
| hSLPI | TCCAGGGAAGAAGAGATGTTGTC (192-214) | TTCCTCCTTGTTGGGTTTGG (275-256) | BC020708 |
| hSPINK5 | TATGAAGCTGTTTGTGGCACAGA (416-438) | ACCCGGTTTTCGCATTCTC (494-476) | NM001127698.1 |
| hElafin | TGTGAAGGCTCTTGCGGG (338-355) | GACCGGCTCCCTCTCACTG (392-374) | BC0109512.1 |
| hSPINK1 | TGTCTGTGGGACTGATGGAAATA (150-172) | TGGCGTTTCCGATTTTCAA (218-200) | BC025790.1 |
| hSerpina1a | TGAACTCACCCACGATATCATCA (1128-1150) | CAGGACGCTCTTCAGATCATAGG (1242-1220) | NM000295.4 |
| hSerpine1 | GCACAACCCCACAGGAACAG (1306-1325) | CCCAGATGAAGGCGTCTTTC (1384-1365) | NM000602.3 |
| mSerpina1a* | TCGATGAGACAGGAACAGAAGCT (1178-1200) | GTGGTCGAAGCGCAGGATAG (1263-1264) | NM009243 |
| mSerpina1b | TCTTTGAAGCCGTTCCTATGTCT (1150-1172) | GGGCTCTGAGTGTGTTCTTCAAAT (1240-1217) | BC012874.1 |
| mSerpina1c | CACAGTCTTACTAGCCGTTCCTTATTCT (1166-1193) | GGGCTCTGAGTGTGTTCTTCAA (1261-1240) | BC021780.1 |
| mSerpina3n | CATCGGGAGTCAGCTATCACA (35-55) | GGTCTTCTTGGACTGCAGCAT (211-191) | NM009252.2 |
| mSerpinb7 | CACTGCCCTGACCCTAATCC (163-182) | AGTGCAGTGCCTTGTCAATCTG (233-212) | Mm.66015 |
